# Utilization of Cerebral Blood Flow Study With Computed Tomography for Subdural Hematoma Management

**DOI:** 10.7759/cureus.16314

**Published:** 2021-07-11

**Authors:** Ryan F Amidon, Christ Ordookhanian, Talia Vartanian, Paul Kaloostian

**Affiliations:** 1 Medicine, Medical College of Wisconsin, Milwaukee, USA; 2 Medicine, University of California, Riverside, Riverside, USA; 3 Physical Medicine and Rehabilitation, University of Southern California, Los Angeles, USA; 4 Neurological Surgery, Riverside Community Hospital, Riverside, USA; 5 Neurological Surgery, Paul Kaloostian M.D. Inc., Riverside, USA

**Keywords:** subdural hematoma, neuroradiology, neuroimaging, radiology, computed tomography, ct perfusion, cerebral blood flow, stroke evaluation, hematoma evaluation, hematoma evacuation

## Abstract

Stroke is among the leading causes of death in the United States, and with our aging population, it will remain a pertinent obstacle in the acute setting. While the field of neuroradiology has advanced tremendously over the years, particularly in improving what we can visualize and quantify, the phrase “time is brain” yet dominates acute stroke management. Optimizing diagnostic protocols for suspected stroke requires a careful balance of data acquisition and speed, as well as taking into account available resources. We present a case of a middle-aged patient with notable risk factors for stroke presenting to the emergency department with altered mental status and suspected stroke. Radiography revealed a large subacute subdural hematoma (SDH) with a mild mass effect on the surface of the brain. The evaluation was supplemented by a computed tomography (CT) and perfusion cerebral blood flow (CBF) study indicating cortical ischemia with penumbra from the SDH compression. SDH evacuation was successfully performed, and patient recovery was achieved within the intensive care unit (ICU). Rapid data acquisition via CBF with CT imaging is crucial for guiding treatment decisions for SDHs. While protocols for ischemic stroke are well-established, SDH protocols are not studied. Thus, we discuss the value of a multimodal CT imaging approach, including CBF studies, in SDH evaluation.

## Introduction

The benefits of optimizing diagnostic approaches for stroke are far-reaching. Each year in the United States, about 800,000 people experience stroke, and it stands as the fifth leading cause of death [1]. Stroke prevalence in adults is 2.5% with seven million Americans of at least 20-years old reporting to have a stroke. Risk factors include hypertension, obesity, diabetes mellitus and hyperglycemia, hyperlipidemia, disorders of heart rhythm, kidney disease, sleep-disordered breathing and sleep duration, smoking/tobacco use, sedentary lifestyle, unhealthy diet, air pollution, social determinants, family history and genetics, and psychosocial factors [1]. Three classes of stroke include ischemic (87% of cases), intracerebral hemorrhagic (ICH) (10%), and subarachnoid hemorrhagic (3%). While ischemic stroke is characterized by a reduction in blood and oxygen delivery to a region of the brain, a hemorrhagic stroke occurs when a blood vessel is ruptured and bleeds.

During an intracerebral hemorrhage (ICH), blood pools in the brain creating a hematoma, which may expand as the bleeding progresses. A large hematoma will cause compression of the brain that may lead to brain damage, neurological complications, and potentially death. Therefore, hematoma expansion is a predictor of greater morbidity and mortality [2]. The phrase “time is brain” perfectly describes the urgency of diagnosis and subsequent treatment. This is highlighted by the finding that the Glasgow Coma Scale (GCS) score of over 20% of ICH patients will drop by at least two points between emergency medical services assessment and evaluation at the emergency department [2]. Subdural hematoma (SDH), as either a complication of ICH or presenting with another etiology, is likewise characterized as a condition with high mortality. In patients requiring surgery, mortality is between 40% and 60%, rising to about 75% in adults over 50-years old at three months post-operation [3]. Alternative causes and risk factors for SDH include head trauma (causing about 70% of cases), antithrombotic therapy, ruptured cerebral aneurysm, cerebral atrophy, tumor, cerebral vascular malformations, vasculopathy, coagulopathy, systemic thrombolysis, and intracranial hypotension [4]. In the stroke world, ICH is known as being particularly fatal and incapacitating [5]. Fortunately, it is far less common than ischemic stroke. Unfortunately, while most centers have established protocols for treating acute ischemic stroke, relatively few have optimized protocols for ICH or SDH management [2].

In this case, we present a middle-aged patient with notable risk factors for stroke presenting to the emergency department with altered mental status. Neuroradiology found evidence of a subacute left SDH with minimal midline shift on CT scan, but with worsening neurological examination. CBF study obtained for stroke management identified this subdural hematoma with evidence of cortical ischemia from the mass effect on the surface of the brain. Hematoma evacuation and subsequent neurological recovery were achieved. Our success highlights the role of multimodal diagnostic imaging studies, including computed tomography perfusion (CTP), for suspected stroke patients in the emergency setting with a specific emphasis on the utilization of CTP for diagnosis and guidance of management of SDH.

## Case presentation

**Figure 1:**
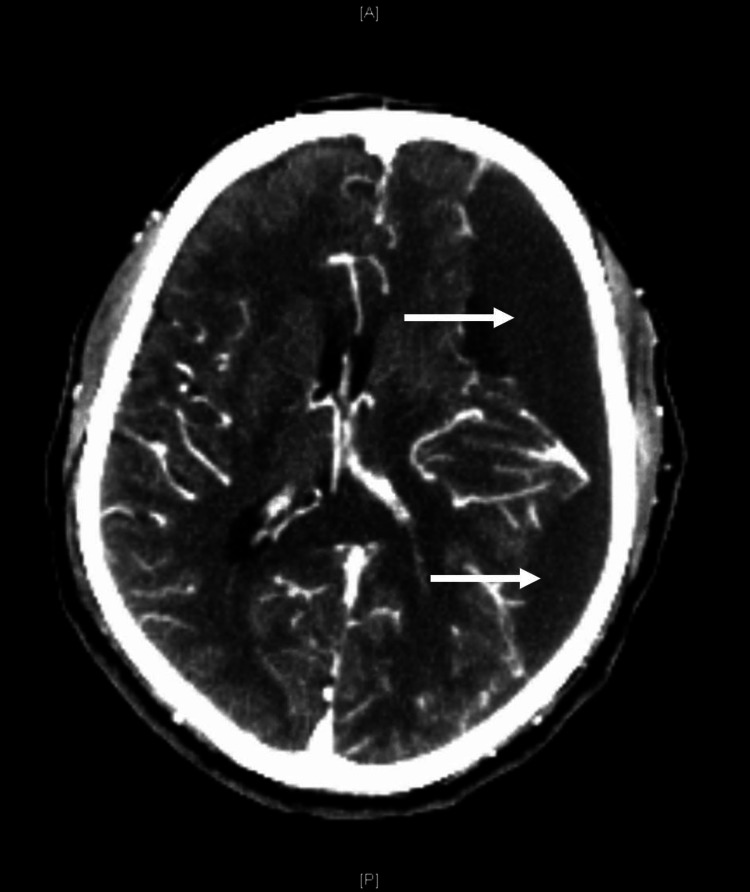
Contrast CT of the head reveals subacute left frontotemporoparietal subdural hematoma. CT: Computed tomography.

A 50-year-old male presented to the emergency department as a code stroke patient with altered mental status upon initial presentation to the healthcare team. Our patient had a past medical history of poorly controlled hypertension and type 2 diabetes mellitus with inconsistent utilization of Lisinopril and metformin. Our patient was not on any statin or anticoagulation use despite a noted elevated atherosclerotic cardiovascular disease (ASCVD) risk score. Initial computed tomography (CT) of the head was significant for a subacute left frontotemporoparietal SDH with 2 mm of midline shift (Figure 1).

**Figure 2:**
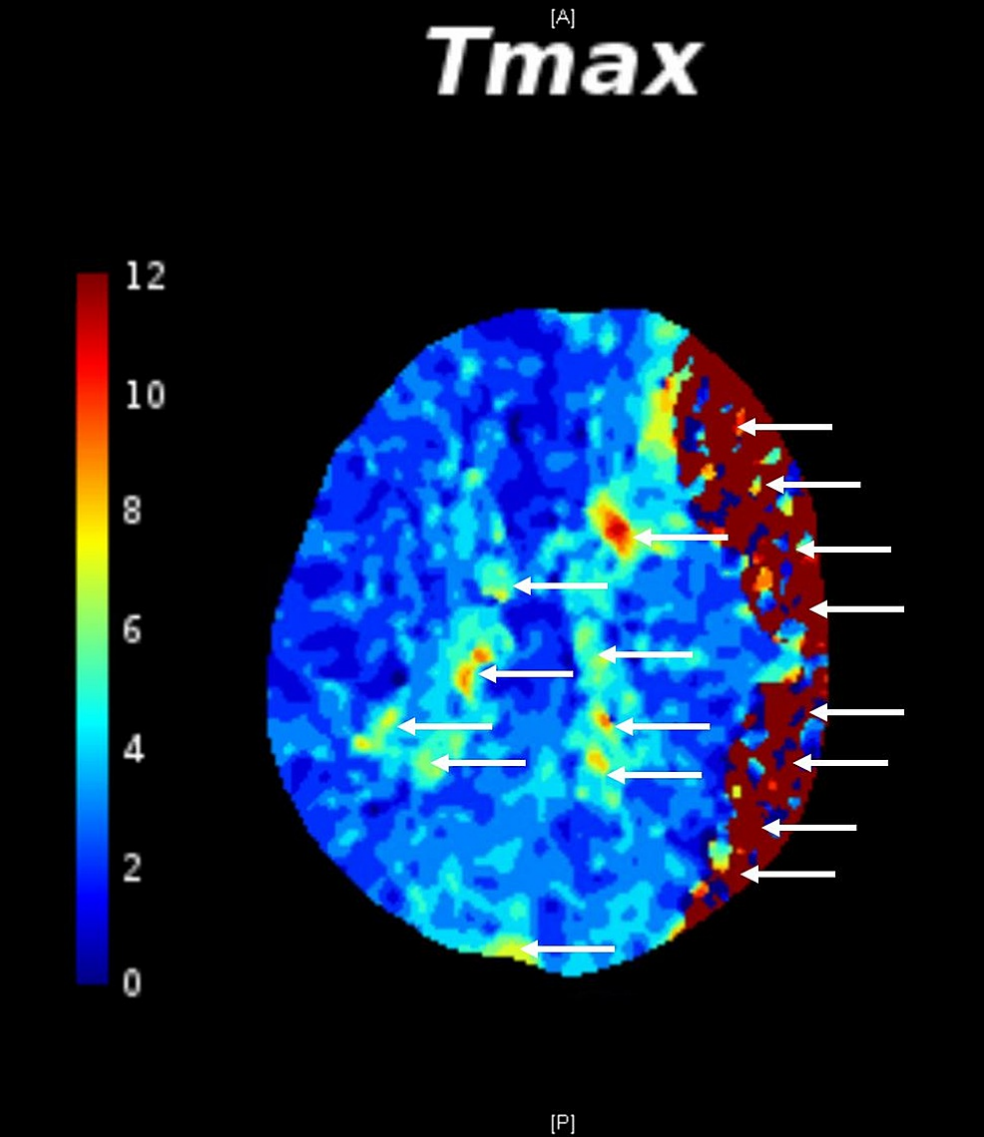
CT cerebral blood flow study confirms subdural hematoma and reveals areas of ischemia/penumbra that are salvageable. CT: Computed tomography.

Per institutional protocol, a cerebral blood flow (CBF) study (CTP) was performed that confirmed the large left SDH with mass effect (Figure 2) as well as ischemia/penumbra on the cortical surface as noted on the CBF imaging (notated as the yellow collar under the subdural hematoma).

**Figure 3:**
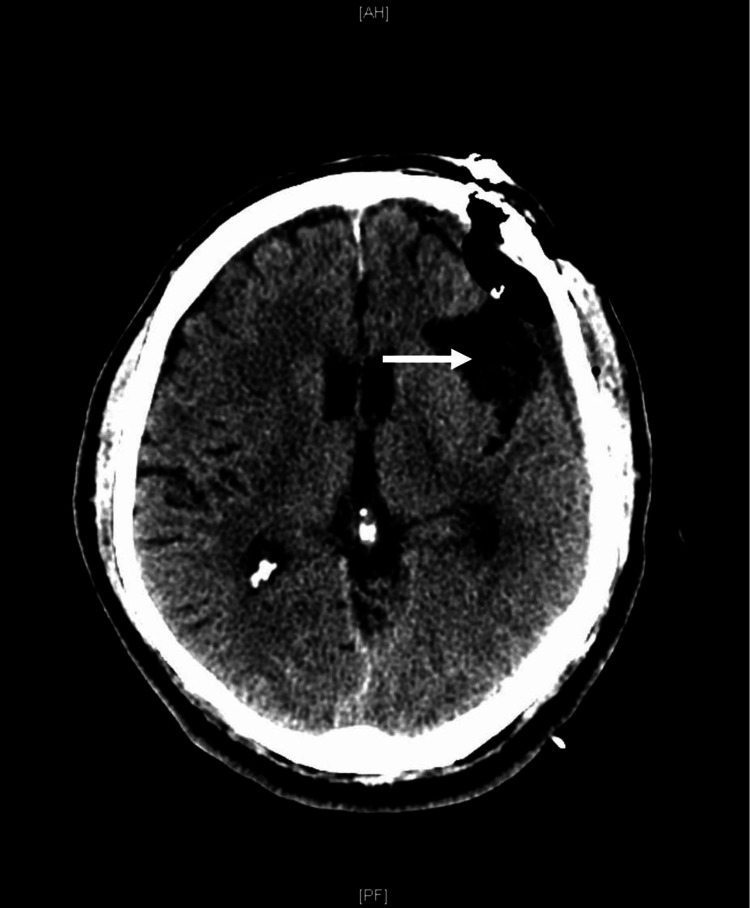
Post-operative CT of head confirms evacuation of subdural hematoma. CT: Computed tomography.

Surgical consultation was requested, and a left craniotomy for SDH evacuation was performed. A post-operative CT scan of the head revealed resolution of the SDH (Figure 3).

In the post-operative realm, our patient recovered well within the intensive care unit (ICU) and was neurologically intact upon transfer to the rehabilitation unit.

## Discussion

The potential for early neurologic decline and the high rate of poor outcomes in the long term support the consensus that aggressive initial treatment is necessary for patients experiencing SDH as a complication of intracerebral hemorrhage (ICH) or from another etiology when injured tissue has the potential to be salvaged before irreversible pathological damage ensues. Before aggressive management can be pursued, an appropriate treatment plan requires sufficient information from diagnostic studies. Clinical characteristics such as vomiting, high systolic blood pressure, headache, coma, and altered mental status are indications of ICH; however, they are not specific to ICH [2]. Other stroke types and SDH alone may manifest with a similar set of symptoms. Our patient’s presentation with altered mental status and history of poorly controlled hypertension and diabetes led to suspicion of stroke. However, confidently distinguishing between ischemic and hemorrhagic stroke, as well as ruling out stroke mimics, required advanced neuroimaging. Fortunately, we were able to identify subacute SDH as a stroke mimic with initial CT. CBF study was completed as well identifying the SDH with indications that the cortical surface of the brain was actively being injured. Additionally, neuroimaging aids help in accurately predicting outcomes to be communicated with the patient’s family when discussing treatment plans as well as to align oneself with realistic outcomes [5].

Neuroimaging modalities vary in the information they provide, accessibility, acquisition time, and limitations. Initial imaging typically involves non-contrast CT (NCCT) to determine whether a stroke is ischemic or hemorrhagic, to rule out stroke mimics, and to guide decisions regarding the use of intravenous tissue plasminogen activator (tPA) as part of reperfusion therapy in cases of ischemic stroke [6,7]. Contrast CT can be used in ICH and hematoma cases to identify patients at high risk of hematoma expansion by visualizing contrast extravasation in the hematoma [2]. While CT angiography (CTA) can supplement contrast CT for evaluating ICH, it is typically used to identify stenosis and occlusion of vessels. Limitations include prior allergic reaction to contrast agents that are unable or of high risk to control with corticosteroids and antihistamines as well as patients with declining renal function. Therefore, baseline creatinine and estimated glomerular filtration rates (eGFR) are established before performing CTA. The study can be completed within one minute. CTP studies can identify stroke and SDH and aid in determining which regions of the brain may be salvageable. Additionally, CTP can guide treatment when the time of stroke onset is unknown or in cases of awakening stroke. Traditionally, CTA in combination with CTP provides quantitative and qualitative data allowing one to distinguish between normal tissue, penumbra, and infarcted core [7]. However, current software allows for quantitative and qualitative data to be obtained from CTP alone.

While different magnetic resonance imaging (MRI) sequences hold unique advantages, traditional MRI studies can take up to an hour to complete and are not as available as CT, making them less common in acute settings. MRI limitations include incompatibility with patients with pacemakers, some metallic implants, allergic reactions to contrast agents, and major claustrophobia [6]. Specialized MRI studies are useful in evaluating patients suspected of having a subarachnoid hemorrhage. MRI diffusion-weighted images (DWI) can improve ischemic stroke detection from 50% to more than 95% once the hemorrhagic stroke is ruled out by CT and can be completed within 10 minutes [6]. It can also aid in excluding stroke mimics. Unfortunately, lack of availability may limit its practical use. Magnetic resonance angiography (MRA) can identify the causes of ischemic stroke as well as underlying aneurysms. MRI perfusion (MRP) studies fulfill the same role as CTP but generate perfusion maps of the entire brain. Positron emission tomography (PET) perfusion studies provide quantitative CBF information, but limited availability and use of short half-life radiotracers make this modality impractical in emergency settings [7]. Digital subtraction angiography can be used to identify and treat numerous types of cerebrovascular disease. Sonography, considered an adjunct modality, can facilitate stroke evaluation in patients who are unstable and/or ineligible for more standard imaging modalities [6].

There are numerous diagnostic modalities that can be used to evaluate patients suspected of having a stroke. Effective neuroimaging protocols will use a combination of modalities that are immediately available; can be completed within a short time frame; and will demonstrate the intracranial arteries, presence and extent of ischemia, and extent of irreversible injury to guide decisions for reperfusion therapy and surgical evacuation, in cases of SDH [7,8]. Therefore, CT modalities remain superior to MRI modalities in the emergency setting as their cumulative data has broad application to stroke and SDH evaluation while being safe and cost-effective [8]. It is also helpful to obtain a serial evaluation of the disease course, rather than images of a single point in time, to observe disease progression [7].

CBF studies, including CTP and MRP, possess unique value in evaluating stroke, stroke-associated SDH, and SDH of alternate etiology. Due to the practical limitations of MRI, we will focus on CTP. Contrast is administered intravenously, and brain sections are repeatedly imaged, tracking the contrast bolus for 60-120 seconds [6,9]. Perfusion maps provide cerebral blood volume (CBV) data, CBF data, and time parameter data, including mean transit time (MTT), time to peak (TTP), and time to maximum (Tmax). MTT is the length of time it takes between arterial inflow and venous outflow; TTP may be used in place of MTT. Tmax refers to the time at which the maximum value of residue function is observed and can indicate a delay in contrast bolus arrival. A value of 0 (at most 4) seconds is expected of normal tissue and blood supply, whereas a greater value represents acute ischemic lesion [7].

During an ischemic stroke, the area of infarct experiences a stark reduction in CBF (less than 30% of normal) and a mild decrease in CBV. This region of CBV loss indicates irreversible damage and is known as the infarct core. The infarct core is also characterized by very high time parameters (MTT, TTP, and Tmax). The surrounding brain tissue, known as the ischemic penumbra, experiences slightly reduced CBF but normal to heightened CBV and is at risk of becoming irreversibly injured unless salvaged. This region has mildly heightened time parameters compared to normal tissue [7]. To maintain homeostasis in response to decreased CBF, the ischemic penumbra’s cerebral autoregulation system dilates collateral blood vessels to raise CBV [6]. CTP can guide decisions regarding reperfusion therapy by demonstrating the extent of irreversibly injured and potentially salvageable tissue [10]. Additionally, CTP provides strong predictors of functional outcome and long-term clinical outcome, including volume of infarct core and the presence of multiple regions of infarct [11,12].

SDH is known to cause ischemic injury from brain compression. The ischemic penumbra is therefore not solely limited to the immediate regions surrounding the hematoma. In cases of ICH-induced hematoma, CTP can identify contrast extravasation (indicating hematoma expansion) not seen in CTA or contrast CT by observing higher perfusion changes, improving the accuracy of outcome predictions [9]. The site of extravasation is known as the spot sign, where there are markedly increased regional CBF and regional CBV values compared to the whole hematoma [13]. In our patient, ischemic lesions were observed in numerous locations along the cortical surface, representing tissue that could potentially be salvaged through surgical intervention. The extent of the injury was quantified through CTP-generated perfusion maps, facilitating estimation of the timeframe of hematoma evacuation. Compression in the brain (seen from the midline shift and mass effect) could have progressed the clinical manifestation from neurological complications to death if left untreated. The decision to perform emergent neurosurgical hematoma evacuation was guided by our neuroimaging findings, paving the way for a successful neurological recovery of our patient.

Multimodal CT imaging, including CTP, can extend the windows for endovascular thrombectomy and intravenous thrombolysis; additionally, CTP can exclusively select patients who respond to reperfusion therapy beyond the six-hour “late window” [14]. Regarding detection of ischemic stroke, Biesbroek et al. found CTP sensitivity to be about 80% with a specificity of 95% [15]. False negatives were the result of either small lacunar infarcts or limited coverage. Kloska et al. similarly found detection sensitivity of about 80% using multimodal CT imaging, including NCCT, CTA, and CTP [16]. Conversely, detection sensitivities of NCCT and CTA alone were about 55% and 58%, respectively. Small infarcts accounted for most false negatives. They also found CBF maps to correlate with the final infarct size with an r2 value of 0.71. Hopyan et al. found that multimodal CT, including NCCT, CTA, and CTP, was about 12% more sensitive than NCCT and CTA and about 18% more sensitive than NCCT alone in stroke determination [17]. Regarding cost-effectiveness, Martinez et al. found that comprehensive CT (NCCT, CTA, and CTP at time of presentation) and comprehensive MR (MRI-DWI, MRA, and MRP) had the greatest lifetime quality-adjusted life-years (QALYs) at 4.81 and 4.82, respectively [18]. When compared with comprehensive CT, the incremental cost-effectiveness ratio of comprehensive MR was $233,000/QALY. While similar in QALYs, comprehensive CT greatly outcompetes comprehensive MR in terms of cost-effectiveness.

Overall, these studies demonstrate the value that CTP studies, and multimodal CT imaging in general, provide to evaluate all patients presenting with clinical signs of stroke. Even when initial imaging reveals SDH as the cause of stroke-like symptoms, CBF studies hold significant value in guiding SDH management by revealing regions of salvageable tissue along the cortical surface or deep surfaces of the brain. Evidence-based neuroimaging protocols should routinely incorporate multimodal CT imaging, including CTP, in SDH evaluation.

## Conclusions

While most centers have established protocols for treating acute ischemic stroke, relatively few have evidence-based neuroimaging protocols for evaluating patients with subacute SDH with minimal midline shift with stroke symptoms in the emergency setting. This case demonstrates that CT with CBF studies should be completed in all patients with SDH in both emergency and non-emergency settings to determine quantitative ischemia/penumbra along the cortical surface or deep surfaces of the brain to determine the necessity and timeframe of surgical hematoma evacuation. A multimodal CT approach, including CTP studies, should be incorporated into subdural hemorrhage evaluation protocols as it provides broad diagnostic information for quantitative effects of the hemorrhage on the brain, is practical for use in an emergency setting, and is the most cost-effective combination of neuroimaging modalities.
